# Definitions of recovery and outcomes of major depression: results from a 10-year follow-up

**DOI:** 10.1111/j.1600-0447.2007.01119.x

**Published:** 2008-01

**Authors:** T A Furukawa, A Fujita, H Harai, R Yoshimura, T Kitamura, K Takahashi

**Affiliations:** 1 Department of Psychiatry and Cognitive-Behavioral Medicine, Nagoya City University Graduate School of Medical Sciences Nagoya, Japan; 2 Division of Clinical Research, Kikuti National Hospital Kumamoto, Japan; 3 Department of Psychiatry, University of Occupational and Environmental Health Kitakyushu, Japan; 4 Department of Clinical Behavioral Sciences (Psychological Medicine), Kumamoto University Graduate School of Medical Sciences Kumamoto, Japan; 5 Aino University Ibaraki, Japan

**Keywords:** depressive disorder, diagnostic criteria, remission, recovery

## Abstract

Furukawa TA, Fujita A, Harai H, Yoshimura R, Kitamura T, Takahashi K. Definitions of recovery and outcomes of major depression: results from a 10-year follow-up.

**Objective:**

Consensus operational definitions for symptomatic remission and recovery of a major depressive episode have been proposed but only irregularly followed.

**Method:**

We examined the predictive validity of different definitions of recovery in a multi-center 10-year follow-up study of an inception cohort of untreated unipolar major depressive episodes (*n* = 95). Time to recovery and time to recurrence after recovery were estimated by Kaplan–Meier survival analyses for alternative definitions requiring 2, 4, 6 or 12 months of remission to declare recovery.

**Results:**

The median time to recovery was 3.0, 4.0, 4.0 and 12.0 months respectively. The index episode lasted longer than 24 months in 9.4%, 9.2%, 12.6% and 24.5%. The median time to subthreshold recurrence was 16.0, 32.0, 42.0 and 74.0 months.

**Conclusion:**

Either 4- or 6-month duration of remission defined a change point before which the episode was continuous and after which the recurrence was reasonably unlikely.

**Significant outcomes**

- Requiring two months of remission is probably too short to declare recovery because a subthreshold recurrence occurs in more than half of the cohort within a year and a half.
- If we require 4 or 6 months before we declare recovery, the median time to recovery is 4 months and that to subthreshold recurrence is nearly 3 years.
- Requiring 12 months of remission before declaring recovery would make the episode discontinuous yet long and inflate the rate of chronicity.

**Limitations**

- The sample size was relatively small and the confidence intervals were accordingly wide.
- This was a naturalistic study and the treatments were not controlled.
- Validity of alternative definitions of remission requires a separate study.

## Introduction

Confusions and inconsistencies persist in the literature with regard to operational definitions of critical change points in the course of a major depressive episode, such as remission, recovery, relapse and recurrence. It was the US NIMH Collaborative Depression Study (CDS), a landmark long-term cohort study of patients with mood disorder, that first operationalized remission as a state with no more than one or two mild depressive criterion symptoms, and recovery as eight or more weeks of remission (1). Some have followed this convention (2–5), while others have not (6–9). But none of these studies has provided empirical examination of the validity of competing definitions.

With regard to the official diagnostic criteria, the definitions in DSM-IV are in line with the CDS definitions because they declare ‘recovery’ if ‘full remission’, which is defined as a period of at least 2 months in which there are not significant symptoms of mania or depression, is attained between two mood episodes (10). The ICD-10 also appears to follow this tradition when it requires ‘at least 2 months free from any significant mood symptoms’ for depressive disorder to be recurrent (11). On the other hand, the DSM-III-R was somewhat aberrant because it required ‘no significant signs or symptoms of the disturbance for at least 6 months’ for an episode to be in full remission, while requiring ‘2 months of return to more or less usual functioning’ for the disorder to be recurrent (12). The DSM-III did not have any explicit definitions for recurrence or remission (13).

This state of confusion is unfortunate not only for the psychiatric sciences because then findings cannot be compared and accumulated, but also for the psychiatric services because then the literature cannot inform the practitioners about how to judge recovery and declare the end of continuation treatment or about whether to recommend maintenance treatment based on knowledge of likelihood of recurrence.

It is important to note that, in this framework for long-term treatment of depression (14, 15), emphasis is placed on the symptomatic aspects of the course of a major depressive episode. This is in line with recent definitions of remission in psychotic disorders (16, 17) and anxiety disorders (18). However, there is growing tendency, especially with regard to schizophrenia and other serious mental disorders, to use the term ‘recovery’ in conjunction with quality and meaning of life in spite of continued symptoms (19). Whether we need to include functional or even broader normalization in the definition of recovery with regard to depression is being discussed and researched (20), but in this article, we follow the symptomatological orientation currently adopted in the mood disorder section of the DSM-IV (10) and ICD-10 (11).

### Aims of the study

The present article therefore sets out to examine the differential predictive validity of the competing definitions of symptomatic recovery. We propose that a more valid definition of recovery should define a change point until which the syndrome is relatively continuous, but after which a return of the syndrome will become reasonably unlikely.

## Material and methods

Data for this report come from the Group for Longitudinal Affective Disorders Study (GLADS), described in detail elsewhere (5, 21). Briefly, it is a multi-center collaborative naturalistic study of patients with heretofore untreated mood episodes who had presented to various psychiatric facilities all over Japan.

The 23 collaborating centers included psychiatric departments of 13 university hospitals and six general hospitals, three mental hospitals and one community mental health center. Participating psychiatrists at each center administered a semi-structured interview, called the Psychiatric Initial Screening for Affective Disorders (PISA) (22) to a representative subset of its first-visit patients to ascertain the patient’s eligibility. The details of the predetermined rules on how to select a subset of first-visit patients were left to individual centers, depending on their human and logistic resources: some centers administered PISA to all their first-visit patients, others did so with those on a certain day of the week and still others did so with those seen by one or two collaborating psychiatrists only. The eligibility criteria were: i) depressive state or manic state; ii) having received no antidepressant or antipsychotic medication in the preceding 3 months; iii) aged 18 years or older; and iv) absence of conditions that would render detailed psychopathological assessment difficult. Out of all the eligible subjects, each participating center was expected to enter the first such patient every 1 or 2 months.

The study protocol was approved by the Ethics Committee of the National Center of Neurology and Psychiatry, Japan, as well as those of the participating centers. Written informed consent was obtained from all participants after full disclosure of the purposes and procedures of the study. The patients eligible for and consenting to the study were then interviewed within 1 week of entry by a psychiatrist using the entry version of the Comprehensive Assessment List for Affective Disorders (COALA) (23). The COALA consists of a series of semi-structured interviews that enable serial assessment of the cohort; these include the entry version, monthly follow-up version, 6-monthly follow-up version and yearly follow-up version. The reliability of the PISA and COALA has been reported to be good to excellent (24). The cohort was followed up monthly until treatment termination, 6-monthly thereafter up to 2 years and then annually up to 10 years. At each assessment, the course of the illness was recorded for each month of the survey period in five grades of 5 = above diagnostic threshold for major depressive episode, 4 = between 5 and 3, 3 = asymptomatic or minimally symptomatic with at most two of nine diagnostic criteria symptoms of at most mild degree, 2 = between 3 and 1 and 1 = above diagnostic threshold for manic episode.

The present paper focuses on the course of the subset of the cohort who were diagnosed with major depressive disorder according to DSM-IV (10). We defined remission in accordance with the NIMH CDS as a state with no more than one or two mild criterion depressive symptoms (2). The CDS then defined recovery as consecutive two months of remission. Once recovery was declared, patients were considered to have fallen into a new mood episode (recurrence) when they met the DSM-IV criteria for major depressive episode, manic episode or hypomanic episode. In addition, if they did not yet meet the criteria for a major depressive episode but had more than two symptoms or had only one or two symptoms that were graver than mild degree for a month, they were considered to have fallen into a ‘subthreshold’ depressive episode. The duration of the well interval was counted after the end of the period required for judging recovery.

The CDS definition of recovery by 2 months of remission has been criticized for being too short (25, 26) and the consensus definitions proposed alternative definitions of recovery by 4 or 6 months of remission. The present paper examines the predictive validity of alternative duration requirements of 2, 4, 6 and 12 months of remission to define recovery. We hypothesized that a more valid definition of duration required for declaring recovery would:

- not prolong the duration of the index episode too much, lest the episode contains too long well periods in itself;
- not increase the rates of chronicity (never attaining recovery) too much, lest we give an overly pessimistic impression that depression is a chronic or incurable disease;
- ensure that the time to recurrence is reasonably long, so that ‘recovery’ once declared can assure the patients that a return of symptoms is reasonably unlikely.

We used the spss for Windows 11.5 (27) to perform Kaplan–Meier analyses to depict survival curves of the major depressive episodes.

## Results

A total of 1853 patients were screened at 23 participating centers between December 1992 and December 1995. A total of 466 patients suffered from broadly defined mood disorders, but either failed to meet the other entry criteria or declined consent and 126 entered the study. Of these, 95 met the DSM-IV criteria for major depressive disorder, either single episode (*n* = 67, 71%) or recurrent (*n* = 28, 29%). Fifty-six subjects (59%) were females, and the mean age was 44.3 (SD 15.2). The mean score for the 17-item Hamilton Rating Scale for Depression was 19.9 (SD 8.6) and 14 (15%) were inpatients upon study entry. The major depressive episode was superimposed on pre-existing dysthymia in five (5%). The median length of episode before study entry was 3.0 months (range: 0.5–48.0).

Table 1 gives the median duration of the index episode and rates of chronicity at 12, 24, 60 and 120 months, depending on the numbers of months of remission required to declare recovery. The table also gives the median time to recurrence of a full episode or a subthreshold episode after recovery was declared, according to each proposed definition.

**Table 1:** Outcomes of major depressive episodes for different definitions of recovery

			Rates of chronicity (%)				Median length of well interval (months)	
				
Definition of recovery	Follow-up rate (%)	Median duration of index episode (months)	12-month	24-month	60-month	120-month	Until full episode recurrence	Until subthreshold recurrence
2 months of remission	90.5	3.0 (2.3–3.7)	16.4	9.4	5.4	3.6	103†	17.0 (1.9–32.1)
4 months of remission	89.5	4.0 (2.9–5.1)	21.9	9.2	6.2	4.1	>101‡	32.0 (1.6–62.4)
6 months of remission	88.4	4.0 (2.2–5.8)	29.9	12.6	6.3	4.2	113 (62–164)	47.0 (10.4–83.6)
12 months of remission	82.1	12.0 (7.9–16.1)	46.0	24.5	12.7	5.1	97†	74.0†

Numbers in parentheses represent 95% confidence intervals.

† 95% confidence intervals could not be calculated.

‡ The cumulative rate of relapse at the latest follow-up of 112 months was 53.2%, so that the median can be estimated to be close to 120.

We illustrate the actual numbers of patients reaching each critical change points or being lost before reaching one in the case of the operational definition of recovery requiring 6 months of remission. Of the original cohort of 95 patients, 84 patients reached recovery so defined, 10 patients were lost to follow-up before reaching recovery and one never experienced recovery over the entire 120 months of follow-up. Of these 84 who were judged recovered, 10 never had a recurrence until the end of the 120-month follow-up, 29 experienced a full episode recurrence, additional 11 experienced a subthreshold recurrence and one presented with a manic episode, 33 were lost to follow-up without ever recording any of these events. Because this was a naturalistic follow-up study and the treatment was not controlled, around the time of recovery, the patients were receiving on average 45.1 (SD 64.7, IQR 0–60) mg/day of imipramine equivalent and only 16 (19%) were on >75 mg/day.

## Discussion

This is the first study to examine the predictive validity of different duration requirements of remission to achieve recovery in terms of the length of the index episode, rates of chronicity and the succeeding well interval until recurrence, based on a long-term naturalistic follow-up data. We found that different definitions can give up to fourfold differences in estimates of episode length and time to subthreshold recurrence but not in time to full recurrence.

Several recent studies have shown that subthreshold depression is associated with psychosocial disability and more severe future course of the illness and requires treatment (28, 29). A systematic review of continuing antidepressant treatment after acute phase treatment reported a consistent relative risk reduction of about 50% in relapse rates up to 3 years (30). For a representative patient in our cohort, then, even if recovery is achieved after 2 months of remission, continuing adequate antidepressant treatment for 1½ years would reduce the subthreshold recurrence rates from 50% to 25%; an average patient may then very well wish to stay on medication. On the other hand, if recovery is declared after 4 or 6 months of remission, one needs to be on medication for 3–4 years to reduce the subthreshold recurrence rates from 50% to 25%; many if not all the patients may choose to stop the medication.

Given our hypotheses regarding the predictive validity of the operational definition of recovery, requiring 12 months would include so much well time before recovery is declared as to draw an unnecessarily chronic picture for the index episode. Requiring only 2 months, on the other hand, would devaluate the significance of recovery because half of the patients so declared would experience a subthreshold recurrence within 1½ years. It is noteworthy that the time to full recurrence remained constant at about 100 months for the three definitions examined. However, given the clinical significance of subthreshold depression noted above, requiring 4- to 6-month remission before declaring recovery appears to be a reasonable definition, as it would not make the index episode unnecessarily chronic, yet assures a relatively low likelihood of subthreshold recurrence once recovery is declared and can provide some indication for ensuing treatments.

There are some possible weaknesses of the present study. First, the sample size was relatively small and 95% confidence intervals were sometimes incalculable or very wide, especially with regard to time to full episode recurrence. This may partly explain the apparent lack of differentiation among various definitions of recovery with regard to this variable. Secondly, this was a naturalistic study, in which we did not control the treatment, and the amount of treatment, actually provided was very low. During the continuation phase, 43% were not receiving any antidepressant therapy and a further 37% were on inadequate treatment with <75 mg/day of imipramine equivalent or <600 mg/day of lithium. Six months later, during the maintenance phase, the corresponding figures were 50% and 29% (31). Thirdly, our cohort consisted mainly of first episode patients and with less severe symptomatology, and this may have influenced estimates of time to recurrence and may be another reason for lack of difference in times to full episode recurrence among various definitions of recovery that we examined. However, Cox regression analyses did not reveal statistically significant influence of recurrent vs. single episode in the median durations of the index episode or the median lengths of the ensuing well intervals. It would be interesting to see analyses similar to ours with the data available from other long-term studies of more severe, recurrent cohorts. Finally, we could not examine the influence of different definitions of remission. One study clearly pointed to the importance of this definition because different symptomatological cut-offs to define remission resulted in an almost sevenfold increase in the length of a depressive episode (32). There are also arguments for including more than symptomatic criteria to define remission (33). These problems need separate examination.

The greatest strength of the current study, on the other hand, is the high follow-up rates achieved through serial assessments of a prospective cohort for 10 years. Some drop-outs were inevitable and we adjusted for the censored cases through survival analyses. The current DSM-IV follows the NIMH CDS tradition and requires 2 months of no significant signs or symptoms of the disturbance before declaring ‘full remission’. The naturalistic follow-up data given in the present report along with the functional and prognostic significance of subthreshold depression demonstrated in cross-sectional and longitudinal studies (28), and the experimental treatment data summarized in the meta-analysis (30) warrant reconsideration of the consensus definitions of remission, recovery, relapse and recurrence in major depression for the upcoming DSM-V.
